# Antimicrobial Activity and Chemical Composition of Essential Oil Extracted from *Solidago canadensis* L. Growing Wild in Slovakia

**DOI:** 10.3390/molecules24071206

**Published:** 2019-03-27

**Authors:** Hazem S. Elshafie, Daniela Gruľová, Beáta Baranová, Lucia Caputo, Laura De Martino, Vincent Sedlák, Ippolito Camele, Vincenzo De Feo

**Affiliations:** 1 School of Agricultural, Forestry, Food and Environmental Sciences University of Basilicata, Viale dell’Ateneo Lucano 10, 85100 Potenza, Italy; hazem.elshafie@unibas.it; 2 Department of Ecology, Faculty of Humanities and Natural Sciences, University of Prešov, 17. Novembra 1, 08001 Prešov, Slovakia; daniela.grulova@unipo.sk (D.G.); beata.baranova@unipo.sk (B.B.); 3 Department of Pharmacy, University of Salerno, I-84084 Fisciano, Italy; lcaputo@unisa.it (L.C.); ldemartino@unisa.it (L.D.M.); defeo@unisa.it (V.D.F.); 4 Department of Biology, Faculty of Humanities and Natural Sciences, University of Prešov, 17. Novembra 1, 08001 Prešov, Slovakia; vincent.sedlak@unipo.sk

**Keywords:** antimicrobial activity, Canadian goldenrod, cell membrane permeability, gas chromatography-mass spectrometry analysis, minimum inhibitory concentration

## Abstract

Plant essential oils (EOs) are one of the most relevant natural products due to their biological, medicinal, and nutritional properties. The promising biological effects of many plants EOs encourage researchers to study their biochemical properties to be used as possible natural alternatives for commercial pesticides and not only as herbal medicines. The current research has been conducted to study the microbicide effect of *Solidago canadensis* L. EO to control some common plant diseases caused by several postharvest phytopathogenic fungi (*Monilinia fructicola*, *Botrytis cinerea*, *Aspergillus niger*, and *Penicillium expansum*) in comparison with Azoxystrobin as a large spectrum fungicide. The antibacterial activity has been carried out against some phytopathogenic bacteria (*Bacillus megaterium* and *Clavibacter michiganensis* (G+ve) and *Xanthomonas campestris, Pseudomonas fluorescens*, and *Pseudomonas syringae* pv. *phaseolicola* (G−ve)) compared to the synthetic antibiotic Tetracycline. Minimum inhibitory concentration was carried out to determine the lowest effective EO dose using a 96-well microplate. The cell membrane permeability was also evaluated by measuring the electric conductivity (EC) to examine the possible mechanisms of action of *S. canadensis* EO. Chemical characterization of EO has been carried out using gas chromatography and mass spectrometry (GC-MS). Thirty-two identified components in *S. canadensis* EO presented 97.7% of total compounds in EO. The principal compounds were identified as germacrene D (34.9%), limonene (12.5%), α-pinene (11.6%), β-elemene (7.1%), and bornyl acetate (6.3%). In addition, *S. canadensis* EO demonstrated promising in vitro antimicrobial activities against the majority of tested phytopathogens at all tested concentrations.

## 1. Introduction

Plant secondary metabolites are characterized by a variety of biological activities. They have been used for their antimicrobial, antioxidant, insecticidal, or phytotoxic effects. Plant essential oils (EOs) have been used in different cultures for medicinal and health purposes due to their antidepressant, stimulating, detoxifying, antimicrobial, antiviral, and calming properties [1,2]. Essential oils have recently gained popularity as natural, safe, and cost-effective substances used in therapy for a number of health concerns [3,4], as well as potential eco-friendly pesticides [5,6].

*Solidago canadensis* L. (*Asteraceae*), commonly known as Canadian goldenrod, is a perennial rhizomatous plant native to North America. It is widely spread in Asia (China, Russia, Japan, and Taiwan), Europe, and Australia, where it is considered as an invasive weed [7,8,9]. The chemical characterization of *S. canadensis* EO has been reported in some publications; in particular germacrene D, α-pinene, and limonene were identified as dominant compounds [10,11,12,13,14,15,16,17,18]. The principal constituents of *S. canadensis* EO are used in phytotherapy for treatment of chronic nephritis, cystitis, urolithiasis, rheumatism, and as an antiphlogistic drug [11,19]. Different kinds of *S. canadensis* extracts of the aerial and underground parts were tested for their antimicrobial activity; in particular, α-pinene, germacrene D, and 6-*epi*-b-cubebene may contribute to the antibacterial ability against *Listeria monocytogenes* and *Staphylococcus aureus* [20,21,22,23,24,25]. The common use of solidago extract as an herbal medicine due to its antioxidant activity encourages the study of its antimicrobial activity, where the oxygenated compounds may contribute to the rapid destruction of the microorganism’s cell wall and increase the cell permeability, which leads to cell death [26].

The novelty of the present study focused on investigation of EO hydrodistilled from invasive species *S. canadensis* and its influence on bacterial strains and fungi, which are considered to be phytopathogenic. This kind of study was presented for the first time.

The main idea of the project is to find a practical use, especially for invasive plant species. These have a negative impact in many areas (ecological, economic, human health, and others). The purpose of this study is to exploit the potential of PEOs in practical applications with the properties of eco-friendly products.

The current research has been carried out to: (i) investigate the chemical composition of *S. canadensis* EO growing in the wild in Slovakia using gas chromatography-mass spectrometry (GC-MS); (ii) evaluate the antimicrobial activity against some post-harvest phytopathogenic fungi (*Monilinia fructicola*, *Botrytis cinerea*, *Aspergillus niger*, and *Penicillium expansum*) and also against some phytopathogenic bacteria (*Bacillus megaterium* and *Clavibacter michiganensis* (G+ve), *Xanthomonas campestris, Pseudomonas fluorescens*, and *Pseudomonas syringae* pv. *phaseolicola* (G−ve), and evaluating the minimum inhibitory concentration (MIC)); and (iii) screen the biological mode of action of *S. canadensis* EO by carrying out a cell membrane permeability assay (CMP).

## 2. Results

### 2.1. Chemical Composition of Solidago Canadensis Essential Oil

The average yield of *S. canadensis* EO of three replicated samples was 0.27 ± 0.05% on dry mass. Thirty-two identified components in *S. canadensis* EO presented 97.7% of the total compounds in EO. The dominant compound was identified as germacrene D (34.9%), followed by limonene (12.5%), α-pinene (11.6%), β-elemen (7.1%), and bornyl acetate (6.3%). The amounts of the other nine components were in the range of 1–3.9%. The order of compounds from the lowest to the highest quantity is camphene < α-caryophyllene < sesquisabinene A < γ-cadinene < epi-bicyclosesquiphellandrene < β-cadinene < β-caryophyllene < β-pinene < β-sabinene (Table 1). The remaining 18 identified components were in amounts of less than 1%. Mono- and sesquiterpene hydrocarbons dominated in *S. canadensis* EO. The percentage of hydrocarbon monoterpenes was 32.9% and hydrocarbon sesquiterpenes was 55.5%. The third group presented oxygenated monoterpenes in an amount of 9.1%.

### 2.2. In Vitro Antifungal Activity

**Figure 1:**
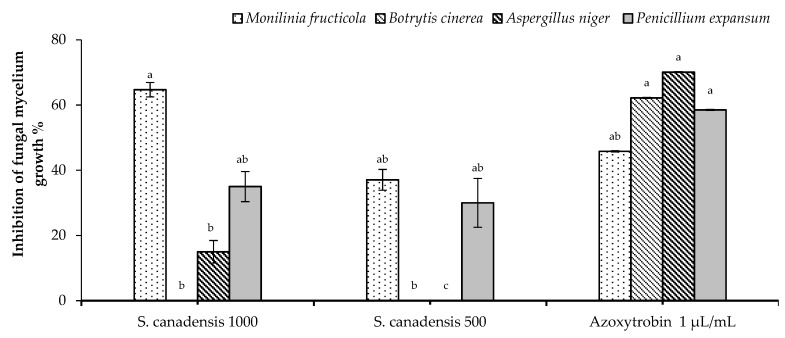
Antifungal activity of *Solidago canadensis* essential oil (EO). Bars with different letters for each tested fungi indicate mean values significantly different at *p* < 0.05 according to *Tukey* B test between *S. canadensis* EO and Azoxystrobin. Data are expressed as mean ± SDs (standard deviations).

*Solidago canadensis* EO showed promising significant antifungal activity against *M. fructicola* and *P. expansum* in a dose dependent manner for both tested concentrations (1000 and 500 µg/mL). In particular, the tested concentration 1000 µg/mL was significantly higher than Azoxystrobin against *M. fructicola*. On the other hand, both tested concentrations were slightly lower than Azoxystrobin against *P. expansum*, whereas, a moderate activity was observed against *A. niger* only at 1000 µg/mL and was significantly lower than Azoxystrobin. No activity against *B. cinerea* for both tested concentrations was noted (Figure 1).

### 2.3. In Vitro Antibacterial Activity

**Figure 2:**
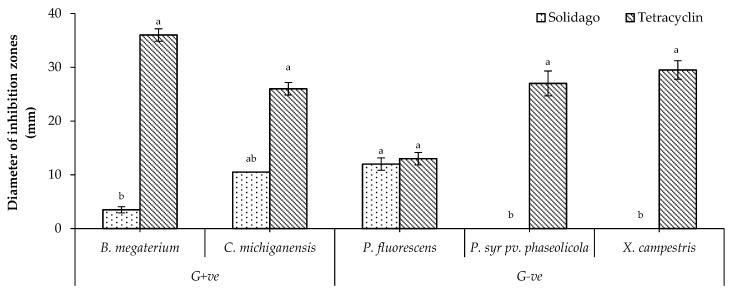
Antibacterial activity of *S. canadensis* EO. Bars with different letters for each tested bacterium indicate mean values significantly different at *p* < 0.05 according to *Tukey* B test between *S. canadensis* EO and Tetracyclin. Data are expressed as mean ± SDs.

*Solidago canadensis* EO showed the highest significant activity against *P. fluorescens* among all tested bacteria, whereas it showed a moderate activity against *C. michiganensis*. In addition, it showed the lowest significant activity against *B. megaterium*. No activity against *P. syringae* pv. *phaseolicola* and *X. campestris* was observed (Figure 2).

### 2.4. Cell Membrane Permeability Assay

In most cases, the main mechanism of fungicide action of any pesticide depends on the destruction of the fungal cell membrane that increases the cell permeability. For that reason, the current assay was carried out to investigate the effect of solidago EO on the CMP of the four tested phytopathogenic fungi treated with different concentrations, measuring their electric conductivity (EC).

**Figure 3:**
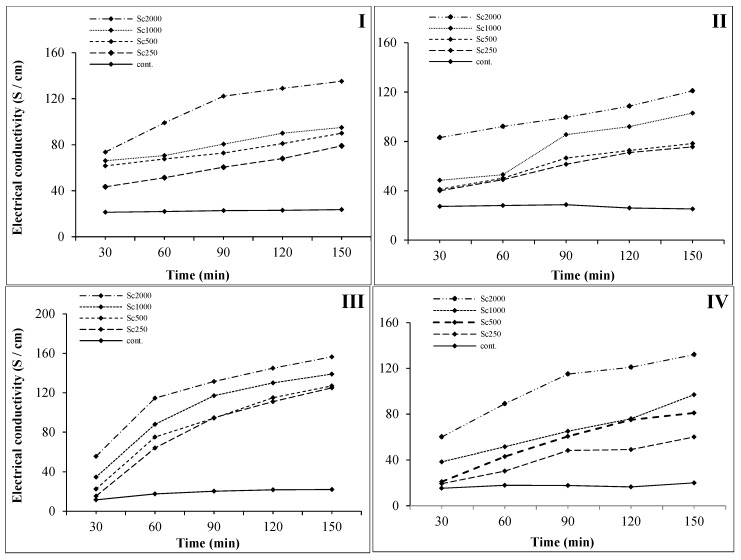
The effect of *S. canadensis* EO on mycelium electrical conductivity of the tested fungi. I: *Botrytis cinerea*; II: *Monilinia fructicola*; III: *Aspergillus niger*; IV: *Penicillium expansum*. Where Sc2000, Sc1000, Sc500, and Sc250 are the tested concentrations of *S. canadensis* EO at 2000, 1000, 500, and 250 µg/mL, respectively. Cont.: control potato dextrose broth (PDB).

Results showed that EC values for *B. cinerea* were raised over time with increased EO concentration (Figure 3-I), whereas the EC values of the *B. cinerea* broth culture (control) were almost stable and ranged between 21.3 and 23.6 S cm^−1^. The EC values of treated cultures with 500 and 1000 µg/mL were much closer to each other and both were slightly higher than the treated culture with 250 µg/mL, whereas the treated culture with 2000 µg/mL showed the highest significant EC value, especially after 90 min of incubation, and this result explained why the initial screening assay with 500 and 1000 µg/mL did not give positive results in plates. Furthermore, *B. cinerea* has a sort of resistance, especially in Agar-nutrient media, in contrast with the initial phase of hyphal formation in broth culture (PDB), where it was delicate and easily damaged. In general, the increasing percentage (IP) of EC value ranged between 204% and 346% after 30 min of incubation and between 334% and 572% after 150 min of incubation for all tested concentrations.

Regarding the changes in mycelia CMP of *M. fructicola* (Figure 3-II), results showed that the EC values of the broth culture (control) were stable until 90 min of incubation and then slightly decreased and ranged between 27.3 and 25.3 S cm^−1^. On the other hand, the increase of EO concentration led to increasing the CMP of the tested fungi in a direct proportional relation, except for 1000 µg/mL, where there was a dramatic increase of the EC after 90 min of incubation. In general, the highest significant EC value was observed in case of the concentration 2000 µg/mL during all incubation period. The IP of EC value ranged between 147% and 304% after 30 min of incubation and between 299% and 478% after 150 min of incubation for the four tested concentrations that ranged between 250 and 2000 µg/mL, respectively.

In the case of *A. niger*, the studied EO led to increasing the CMP of the tested fungi for all tested concentrations in a direct proportional relation for all four tested concentrations (Figure 3-III). The results illustrated that the CMP of *A. niger* also suddenly increased after 60 min for all tested concentrations and then the values tended to be stable until 150 min of incubation. On the other hand, EC values of the broth culture (control) were almost stable along the incubation period, slightly increasing after 60 min of incubation and ranging between 11.5 and 20 S cm^−1^. Low significant differences were observed between the two EO concentrations of 250 and 500 µg/mL after 90 min of incubation. The IP of EC value ranged between 133% and 483% after 30 min of incubation and between 568% and 711% after 150 min of incubation for all tested concentrations that ranged between 250 and 2000 µg/mL, respectively.

In the case of *P. expansum*, the EO concentration of 2000 µg/mL demonstrated the highest significant EC value compared to all other treatments (Figure 3-IV). The results also explained that there was a sharp increase of the CMP of *P. expansum* after 90 min. On the other hand, EC values of the broth culture (control) were stable along the incubation period and ranged between 15.3 and 20 S cm^−1^. Low significant differences were observed between the two EO concentrations of 500 and 1000 µg/mL. The IP of EC value ranged between 126% and 392% after 30 min of incubation and between 300% and 660% after 150 min of incubation for all tested concentrations that ranged between 250 and 2000 µg/mL, respectively.

In conclusion, the highest significant IP of the CMP due to the application of *S. canadensis* EO was measured as 711% and 660% in case of *A. niger* and *P. expansum*, respectively, after 150 min of incubation.

### 2.5. Determination of Minimum Inhibitory Concentration (96-Well Microplate Method)

Minimum inhibitory concentration (MIC), the lowest concentration of tested *S. canadensis* EO that definitely inhibits the growth of *M. fructicola*, was determined by monitoring the absorption of each tested concentration, which is closes to the absorbance of PDB (control) 0.15 with an error margin not exceeding 0.01.

In this regard, results demonstrated that all tested concentrations except 800 µg/mL could completely inhibit the growth of *M. fructicola* after 7 days of incubation at 24 ± 2 °C. In particular, the tested concentration at 1600 µg/mL showed the highest significant inhibition after 4 days with absorbance of 0.21 ± 0.01 (Table 2). In the case of 1400 µg/mL, the highest significant inhibition was observed after 6 days of incubation with absorbance of 0.25 ± 0.05 (Table 2). The two tested concentrations (1000 and 1200 µg/mL) achieved complete fungal growth inhibition after 7 days of incubation and their absorbances were measured as 0.22 ± 0.03 and 0.21 ± 0.03, respectively (Table 2). On the other hand, the lowest tested concentration (800 µg/mL) did not show any growth inhibition, even after 7 days of incubation (Table 2). The MIC of the tested concentration (800 µg/mL) may reach the complete inhibition of the fungal growth after more than 6 days of incubation, because after that period the fungus still grew normally.

## 3. Discussion

The EO isolated from the dry mass of *S. canadensis* generally yielded in the range of 0.21–0.34% [14], while in comparison with the EO yield from different plant parts, a higher amount was found in inflorescence (0.35–1.47%) [15,18,28] than in other aerial parts (0.11–0.16%) [15]. Composition of EO depends on ecological and climatic conditions, the ontogenesis phase, as well as from the processing within the harvest and method of isolation, and generally the yield of EO increase with plant maturation [10,18,29]. Similarly with our identification, the germacrene D was also considered as a dominant compound within the range 19.8–39.2% [10,15,17,18]. On the other hand, β-cubebene (26.9%) was identified as the main compound of *S. canadensis* by Kasali et al. [30]. In all cases, the main compound was followed by α-pinene (2.9–28.1%), limonene (5–11.5%), β-pinene (2.1–9.3%), and bornyl acetate (3.2–9.2%).

Different compositions of EO were identified in the roots of *S. canadensis*. The dominant compound was identified as thymol (20.25 %), followed by α-copaene (6.26%) and carvacrol (5.51%) [12]. Mono- and sesquiterpenes have been identified among the main dominant hydrocarbon groups in *S. canadensis* EO, as was also identified in our sample [15,18].

The antimicrobial activity of S*. canadensis* EO is mainly due to its high content of active secondary metabolites, such as flavonoids, terpenoid, phenolic compounds, and polysaccharides [24,31]. There are few conducted research studies about the biological effect of the vegetal extract and EO of *S. canadensis*, such as Deepa and Velayutham [32], who reported that different extracts of *S. canadensis* showed promising antibacterial activity against some pathogenic bacteria, such as *Salmonella typhi*, using disc diffusion method (in vitro) compared to ciprofloxacin, a commercial antibiotic.

In particular, the obtained results of antifungal activity and CMP assays are in agreement with Liu et al. [24] who studied the effect of solidago EO on the hyphal morphology and cell ultrastructure of *B. cinerea* by using the method described by Yu et al. [33]. The hyphae of *B. cinerea* became more shrunken and thinner after treatment with *S. canadensis* EO and its cell wall structure was damaged, hence the exosmosis increased as it was scanned by electron and transmission electron microscope [24]. This mechanism is highly related to our hypothesis of the biological mode of action of *S. canadensis* EO, which is due to cell wall damage of the fungus and increasing the CMP, ending with complete fungal death.

On the other hand, the MIC of solidago EO ranged between 1000 and 1600 µg/mL against *M. fructicola* during an incubation period starting from 4 days until 7 days. These results also showed that solidago EO can remain efficient in contact with serious post-harvest phytopathogens even at a low concentration (1 µg/µL) and can achieve promising antifungal activity during at a maximum period of 7 days at room temperature.

## 4. Conclusions

Under the strict EU legislation, there is an annual increase of the prohibited substances used in crop protection in agriculture. At the same time, there is a big challenge to develop new products that are both organic and safe for the health of living organisms. The current research evaluated possible antimicrobial activity of EO hydrodistilled from the invasive plant species *S. canadensis* and investigate its mechanism of action. One of the ways how to deal with the invasive species is to investigate their possible agricultural or ecological application instead of weeding them out. Based on the results, significant microbicidal effect against some phytopathogens (bacteria and fungi) was observed. Natural-based products are usually simple to prepare, are not financially demanding, and because of their natural origin they are environmentally acceptable which could be related to the investigated EO from *S. canadensis*.

## 5. Materials and Methods

### 5.1. Plant Material

Aboveground plant samples (stems with leaves and inflorescence) at full-bloom stage were randomly selected and collected in August 2016 from the locality Ľubotice (49°0′13″ N, 21°16′21″). Species were characterized according to Cvachová and Gojdičová’s method [34]. The plant material was dried at room temperature in a thin layer on filter paper for about 14 days. The drying was done until the material reached a constant dry weight. The sample was stored as a voucher specimen SC2016-UNIPO224 at the Department of Ecology, University of Presov.

### 5.2. Isolation of Essential Oil

Twenty grams of the sample of *S. canadensis* was ground in a blender and then hydrodistilled in a Clevenger-type apparatus for 2 h. The oils were solubilized in n-hexane and stored under N_2_ at +4 °C in dark until analysis. The oil yield of the plant materials was a colorless-yellow oil. Pure EO for analysis by GC-MS was diluted to 1:1000 ratios in *n*-hexane. Analyses were replicated three times.

### 5.3. Gas-Chromatography-Mass Spectrometry Analysis of Essential Oil

The GC-MS analyses were carried out on a Varian 450-GC connected with a Varian 220-MS. Separation was achieved using a Bruker capillary column: Br 5ms (30 m× 0.25 mm i.d., 0.25 μm film thickness). Injector type 1177 was heated to a temperature of 220 °C. Injection mode was splitless (1 μL of a 1:1000 *n*-hexane solution). Helium was used as a carrier gas at a constant column flow rate of 1.2 mL/min. Column temperature was programmed—initial temperature was 50 °C for 10 min, then increased to 100 °C at 3 °C/min, was maintained as isothermal for 5 min, and then increased to 150 °C at 10 °C/min. The total time for analysis was 87.67 min. Analyses were also run with the same operating conditions by using an apolar DB-5 fused silica capillary column (30 m × 0.25 mm, 0.25 μm film thicknesses). In both cases, helium was used as the carrier gas (1.2 mL/min). The mass spectrometer trap was heated to 200 °C, manifold 50 °C, and transfer line 270 °C. Mass spectra were scanned every 1 s in the range 40–650 m/z.

Most constituents were identified by comparison of their Kovats retention indices (Ki), with those of the authentic compounds available in our laboratories. The Kovats retention indices were determined in relation to the retention time (Rt) values of an homologous series of *n*-alkanes (C10–C35). Further identification was made by comparison of the mass spectra with either those stored in NIST 02 library or with those from the literature [27]. Components’ relative concentrations were obtained by peak area normalization. No response factors were calculated.

### 5.4. Antifungal Activity

#### 5.4.1. Tested fungal isolates

The tested phytopathogenic fungi were monoconidic isolates *M. fructicola* (G.Winter) Honey (P1605 from plum), *B. cinerea* Pers. (S1132 from strawberry), *A. niger* van Tieghem (G1008 from grape), and *P. expansum* Link (A333 from apple), previously identified based on their microscopic morphological features and molecular methods based on polymerase chain reaction (PCR). The amplicons obtained were directly sequenced and compared with those available in GenBank nucleotide archive using Basic Local Alignment Search Tool software (BLAST-USA) [35]. They were stored at 4 °C as pure cultures in the mycotheca of the School of Agricultural, Forestry, Food, and Environmental Sciences of Basilicata University, Potenza, Italy. The fungal species were cultured on potato dextrose agar (PDA) at 24 ± 2 °C.

#### 5.4.2. Fungicidal assay

The possible fungicidal activity of the studied solidago EO was evaluated following the method of Soylu et al. [36] and Elshafie et al. [37] at two different concentrations (1000 and 500 µg/mL), diluted in 0.2% Tween-20 and then incorporated into potato dextrose agar (PDA) medium at 45 °C. Fungal disk (0.5 cm) from 96 h fresh culture was inoculated in the center of each Petri dish. All plates were incubated at 22 °C for 96 h in darkness conditions and the diameter of the fungal mycelium was measured in mm. The PDA plates without any treatment were inoculated with fungal disks as control. The fungitoxicity was expressed as percentage of growth inhibition (PGI) and calculated according to the formula of Zygadlo et al. [38] (Equation (1)) in comparison with Azoxystrobin, a large spectrum fungicide, as control incorporated at 0.8 µL/mL to PDA nutrient medium according to the international limit of microbicide standards:

(1) $$\mathrm{PGI}\left(\%\right)=\frac{100\times \left(\mathrm{GC}-\mathrm{GT}\right)}{\mathrm{GC}}$$

where PGI is the percentage of growth inhibition, GC is the average diameter of fungal mycelium in PDA (control), and GT is the average diameter of fungal mycelium on the EO-treated PDA dish.

### 5.5. Antibacterial Activity

#### 5.5.1. Tested Bacterial Strains

Two G+ve bacterial strains (*B. megaterium* de Bary ITM100 and *C. michiganensis* Smith) and three G−ve bacterial strains (*P. fluorescens* Flügge (Migula), *P. syringae* pv. *phaseolicola* Van Hall, and *X. campestris* Pammel) were tested. All bacterial isolates were identified based on PCR amplification, gene sequencing, and Blast analysis [35]. All studied strain conserved in the collection of the School of Agricultural, Forestry, Food, and Environmental Sciences of Basilicata University, Potenza, Italy.

#### 5.5.2. Bactericidal Assay

The antibacterial test of the studied EO was carried out using disc diffusion method of Bhunia et al. [39] with some modifications, as reported by Elshafie et al. [40], using king B (KB) and nutrient agar (NA) media [41,42]. A bacterial suspension of each tested bacteria was prepared in sterile distilled water adjusted at 10^6^ colony forming unit per milliliter (CFU/mL) (OD ≈ 0.2 nm). Then, an aliquot of soft agar (0.7%) and bacterial suspension (9:1; *v/v*) was prepared and 4 mL was added over each plate (90 mm, diameter), containing 10 mL of a nutrient medium. Blank Discs (6 mm)-OXOID were placed after that over KB-plate surfaces and about 15 µL of the tested EO (20%) diluted in distilled water and 0.2% Tween-20 was carefully applied over blank discs. The antibacterial activity was estimated by measuring the diameter of the inhibition zone (mm) formed around each treated point compared to tetracycline (1600 µg/mL), according to the international limit of microbicide standards.

### 5.6. Cell Membrane Permeability

Cell membrane permeability was determined by measuring EC. Five mycelial disks, taken from the plates of four tested pathogenic fungi (*M. fructicola*, *A. niger*, *P. expansum*, and *B. cinerea*) were transferred into liquid PDB medium and cultured for 3 days at 28 °C and 175 rpm. One gram of mycelia was blended with 20 mL of 250, 500, 1000, and 2000 μg/mL EO and stored at room temperature (20 ± 2 °C). The EC values of the solutions were determined after 30, 60, 90, 120, and 150 min of incubation. The IP of the EC value has been measure using the following equation:

(2) $$\mathrm{IP}=\frac{E.Ct}{E.C\mathrm{ctrl}}\times 100$$

where E.Ct is the EC value of the treated sample and E.C ctrl is the EC value of the PDB broth culture.

### 5.7. Determination of Minimum Inhibitory Concentration (96-Well Microplate Method)

The minimum inhibitory concentration (MIC) has been carried out against the most inhibited pathogenic fungi using *96-well* microplates (Nunc MaxiSorp®, Vedbaek, Denmark) by a micro-dilution method, as reported by Elshafie et al. [43,44]. A 4 mL liquid suspension from fresh fungal cultures (96 h) was prepared at 10^8^ spore/mL. The tested EO was dissolved in potato dextrose broth (PDB) at 800, 1000, 1200, 1400, and 1600 µL/mL according to the obtained results from the initial antifungal screening assay. Two hundred µL/well from each prepared concentration of EO and 100 µL/well of the prepared fungal suspension were added in the microplate and then incubated at 24 ± 2 °C. The absorbance was measured at λ = 450 nm using an Elisa microplate reader instrument (DAS s.r.l., Rome, Italy) after 48, 120, and 168 h. The whole experiment was repeated in triplicate.

### 5.8. Statistical Analysis

Results obtained from the current research were statistically processed and subjected to analysis of variance one-way ANOVA, followed by *Tukey* B Post Hoc multiple comparison test with a probability of *p* < 0.05, using statistical package for the social sciences (SPSS) version 13.0 (Prentice Hall, Chicago, IL, USA, 2004) to detect any significant difference in behavior of the tested EOs against the tested microorganisms.

## Figures and Tables

**Table 1:** Chemical composition of *Solidago canadensis* essential oil (EO) using gas chromatography-mass spectrometry (GC-MS).

No.	Compound Name	[%]	Ki	Ki lit.	Identif.
1.	α-Pinene	11.6	922	936	Ki; S; MS
2.	Camphene	1.0	943	950	Ki; MS
3.	Sabinene	3.9	964	973	Ki; MS
4.	β-Pinene	3.1	972	978	Ki; S; MS
5.	α -Phellandrene	0.3	997	1002	Ki; MS
6.	m-Cymene	0.1	1013	1013	Ki; MS
7.	Limonene	12.5	1014	1025	Ki; S; MS
8.	β-trans-Ocimene	0.4	1034	1029	Ki; MS
9.	γ-Terpinen	0.1	1047	1051	Ki; S; MS
10.	Cyclohexane, 2-ethenyl-1,1-dimethyl-3-methylene-	0.2	1071		Ki; MS
11.	α-Campholenal	0.2	1106	1105	Ki; MS
12.	trans-Pinocarveol	0.2	1131	1126	Ki; MS
13.	trans-Verbenol	0.1	1136	1136	Ki; MS
14.	1-Terpinen-4-ol	0.1	1137	1137	Ki; MS
15.	3-Thujene-10-al	0.2	1158	1158	Ki; MS
16.	Myrtenal	0.5	1175	1172	Ki; MS
17.	Carveol	0.1	1188	1200	Ki; MS
18.	Verbenone	0.1	1204	1183	Ki; MS
19.	trans-Carveol	0.3	1207	1210	Ki; MS
20.	Carvone	0.2	1210	1214	Ki; S; MS
21.	Bornyl acetate	6.3	1270	1270	Ki; S; MS
22.	a-Terpinyl acetate	0.8	1334	1335	Ki; MS
23.	β-Cubebene	0.2	1355	1355	Ki; MS
24.	α-Copaene	0.4	1376	1379	Ki; MS
25.	β-Elemen	7.1	1387	1389	Ki; MS
26.	β-Caryophyllene	3.0	1421	1421	Ki; S; MS
27.	Sesquisabinene A	1.5	1435	1435	Ki; MS
28.	α-Caryophyllene	1.1	1456	1454	Ki; MS
29.	Epi-bicyclosesquiphellandrene	2.5	1470	1487	Ki; MS
30.	Germacrene D	34.9	1480	1480	Ki; S; MS
31.	γ-Cadinene	2.1	1507	1507	Ki; MS
32.	β-Cadinene	2.8	1526	1526	Ki; MS
	Total	97.7			
	Hydrocarbon monoterpenes	32.9			
	Oxygenated monoterpenes	9.1			
	Sesquiterpene hydrocarbons	55.5			
	Oxygenated sesquiterpenes	0.0			
	Others	0.2			

Note: Ki = Kovat’s retention index compared between software prediction (Ki) and literature (Ki lit.) [27]. Identification of compounds: Ki- Kovat’s retention index, S-standard co-injection, MS = mass spectrometry.

**Table 2:** Minimum inhibitory concentration (MIC) of solidago EO against *Monilinia fructicola*.

	Absorbance of Fungal Mycelium Growth at 450 nm				
	3 days ^b^	4 days	5 days	6 days	7 days
**PDB + F**	0.03 ± 0.02c	1.31 ± 0.25b	1.40 ± 0.25b	1.58 ± 0.32a	1.63 ± 0.32a
**800 µg/mL ^a^**	0.03 ± 0.00b	0.71 ± 0.05a	0.75 ± 0.05a	0.74 ± 0.15a	0.76 ± 0.17a
**1000 µg/mL**	0.02 ± 0.00c	0.46 ± 0.10b	0.51 ± 0.05b	0.48 ± 0.03b	* 0.22 ± 0.03a
**1200 µg/mL**	0.02 ± 0.00c	0.35 ± 0.05b	0.38 ± 0.04b	0.44 ± 0.02b	* 0.21 ± 0.03a
**1400 µg/mL**	0.02 ± 0.00c	0.36 ± 0.04b	0.35 ± 0.07b	* 0.25 ± 0.05a	0.22 ± 0.03a
**1600 µg/mL**	0.01 ± 0.00b	* 0.21 ± 0.01a	0.18 ± 0.03a	0.14 ± 0.05ab	0.15 ± 0.05ab
**PDB**	0.00 ± 0.00	0.15 ± 0.0	0.15 ± 0.0	0.15 ± 0.0	0.16 ± 0.0

Values were recorded as the mean absorbance at 450 nm (three replicates) ± SDs. Values followed by different letters in each horizontal row were significantly different according to *Tukey* B test at *P* < 0.05. (a) The tested concentration of solidago EO ranged between 800 and 1600 µg/mL; (b) the incubation period (days); (*) the MIC for each tested concentration at each incubation time. PDB + F: potato dextrose broth inoculated with *M. fructicola* (positive control). PDB: potato dextrose broth (negative control).
